# Compressive Sensing Inference of Neuronal Network Connectivity in Balanced Neuronal Dynamics

**DOI:** 10.3389/fnins.2019.01101

**Published:** 2019-10-17

**Authors:** Victor J. Barranca, Douglas Zhou

**Affiliations:** ^1^Department of Mathematics and Statistics, Swarthmore College, Swarthmore, PA, United States; ^2^School of Mathematical Sciences, Shanghai Jiao Tong University, Shanghai, China; ^3^Ministry of Education Key Laboratory of Scientific and Engineering Computing, Shanghai Jiao Tong University, Shanghai, China; ^4^Institute of Natural Sciences, Shanghai Jiao Tong University, Shanghai, China

**Keywords:** neuronal networks, balanced networks, signal processing, network dynamics, connectivity reconstruction

## Abstract

Determining the structure of a network is of central importance to understanding its function in both neuroscience and applied mathematics. However, recovering the structural connectivity of neuronal networks remains a fundamental challenge both theoretically and experimentally. While neuronal networks operate in certain dynamical regimes, which may influence their connectivity reconstruction, there is widespread experimental evidence of a balanced neuronal operating state in which strong excitatory and inhibitory inputs are dynamically adjusted such that neuronal voltages primarily remain near resting potential. Utilizing the dynamics of model neurons in such a balanced regime in conjunction with the ubiquitous sparse connectivity structure of neuronal networks, we develop a compressive sensing theoretical framework for efficiently reconstructing network connections by measuring individual neuronal activity in response to a relatively small ensemble of random stimuli injected over a short time scale. By tuning the network dynamical regime, we determine that the highest fidelity reconstructions are achievable in the balanced state. We hypothesize the balanced dynamics observed *in vivo* may therefore be a result of evolutionary selection for optimal information encoding and expect the methodology developed to be generalizable for alternative model networks as well as experimental paradigms.

## 1. Introduction

The connectivity of neuronal networks is fundamental for establishing the link between brain structure and function (Boccaletti et al., 2006; Stevenson et al., 2008; Gomez-Rodriguez et al., 2012); however, recovering the structural connectivity in neuronal networks is still a challenging problem both theoretically and experimentally (Salinas and Sejnowski, 2001; Song et al., 2005; Friston, 2011; Kleinfeld et al., 2011; Bargmann and Marder, 2013). Recent experimental advances, such as diffusion tensor imaging (DTI), dense electron microscopy (EM), and highly resolved tracer injections, have facilitated improved measurement of network connectivity, but constructing complete neuronal wiring diagrams for networks of thousands or more neurons is currently infeasible due largely to the small spatial scale and the dense packing of nervous tissue (Lichtman and Denk, 2011; Sporns, 2011; Briggman and Bock, 2012; Markov et al., 2013). Likewise, modern mathematical approaches for recovering network connectivity based on measured neuronal activity, such as Granger causality, information theory, and Bayesian analysis, typically demand linear dynamics or long observation times (Aertsen et al., 1989; Sporns et al., 2004; Timme, 2007; Eldawlatly et al., 2010; Friston, 2011; Hutchison et al., 2013; Zhou et al., 2013b, 2014; Goñi et al., 2014). Is it possible to achieve the successful reconstruction of network connectivity from the measurement of individual non-linear neuronal dynamics within a short time scale?

To address this central question, we develop a novel theoretical framework for the recovery of neuronal connectivity based on both network sparsity and balanced dynamics. Sparse connectivity among neurons is widely observed on large (inter-cortical) and small (local circuit) spatial scales (Mason et al., 1991; Markram et al., 1997; Achard and Bullmore, 2007; He et al., 2007; Ganmor et al., 2011), and, therefore, the amount of observed activity required to reconstruct network connectivity may be significantly smaller than suggested by estimates using only the total network size. *Compressive sensing* (CS) theory has emerged as a useful methodology for sampling and reconstructing sparse signals (Candes et al., 2006; Donoho, 2006; Gross et al., 2010; Wang et al., 2011b) and has primarily been utilized in estimating the connectivity of linear or time-invariant network models (Hu et al., 2009; Mishchenko and Paninski, 2012). In the case of realistic neuronal networks, their non-linear dynamics in time pose a major conceptual difficulty, particularly in isolating the impact of direct network connections on recorded activity from the effects of indirect neuronal interactions and the external drive.

We demonstrate that the reconstruction of neuronal connectivity based on compressive sensing of non-linear network dynamics is indeed possible in an appropriately *balanced dynamical regime* in which fluctuations in neuronal input largely drive firing events. Numerous experimental studies demonstrate that neuronal firing events are generally irregular, with large excitatory and inhibitory inputs dynamically balanced such that the voltage of a neuron typically resides near the resting potential for a broad class of external stimulation (Britten et al., 1993; Shadlen and Newsome, 1998; Compte et al., 2003; Haider et al., 2006; Tan and Wehr, 2009; London et al., 2010; Runyan et al., 2010; Isaacson and Scanziani, 2011; Xue et al., 2014). Theoretical work corroborates the existence of this operating regime for balanced network models in which neurons are sparsely connected while strongly coupled, such that neuronal activity is highly variable and heterogeneous across the network (van Vreeswijk and Sompolinsky, 1996, 1998; Troyer and Miller, 1997; Vogels and Abbott, 2005; Miura et al., 2007; Mongillo et al., 2012). Here, we utilize the same binary-state network model as such previous studies and demonstrate that using a small ensemble of random inputs and corresponding time-averaged measurements of neuronal dynamics collected over a short time scale, it is possible to achieve high fidelity reconstructions of recurrent connectivity for sparsely connected networks of excitatory and inhibitory neurons. We show that the quality of this reconstruction improves as the network dynamics are further balanced, expecting that for physiological networks, once in the balanced state, CS-based estimates of network connectivity are feasible. We hypothesize that the balanced operating regime may have arisen in sensory systems from evolution as a means of optimally encoding both connectivity and stimulus information through network dynamics.

## 2. Results

### 2.1. Compressive Sensing of Balanced Dynamics

To investigate the reconstruction of neuronal network connectivity in the balanced state, we consider a mechanistic binary-state model with non-linear dynamics (van Vreeswijk and Sompolinsky, 1996, 1998). The model network is composed of *N* neurons, such that *N*_*E*_ neurons are excitatory (E) and *N*_*I*_ neurons are inhibitory (I). The state of the *i*th neuron in the *k*th population (*k* = *E, I*) at time *t* is prescribed by

(1) $$\begin{array}{r} \sigma_{k}^{i}\left(t\right)=H\left(\mu_{k}^{i}\left(t\right)-\theta_{k}\right), \end{array}$$

where *H*(·) denotes the Heaviside function and θ_*k*_ is the firing threshold for the neurons in population *k*. The total synaptic drive $\mu_{k}^{i}\left(t\right)$ into the *i*th neuron in the *k*th population at time *t* is

(2) $$\begin{array}{r} \mu_{k}^{i}\left(t\right)=\overset{N_{E}}{\underset{j=1}{\sum }}R_{kE}^{ij}\sigma_{E}^{j}\left(t\right)+\overset{N_{I}}{\underset{j=1}{\sum }}R_{kI}^{ij}\sigma_{I}^{j}\left(t\right)+{\left(\text{F}\text{p}\right)}_{k}^{i}, \end{array}$$

where $R_{kl}^{ij}$ denotes the connection strength between the *i*th post-synaptic neuron in the *k*th population and the *j*^th^ pre-synaptic neuron in the *l*^th^ population (*l* = *E, I*), and ${\left(\text{F}\text{p}\right)}_{k}^{i}$ is the total external input into the *i*th neuron in the *k*th population. The connection strength $R_{kl}^{ij}$ is chosen to be $R_{kl}/\sqrt{K}$ with probability *K*/*N*_*l*_ and 0 otherwise. In this case, the excitatory connection strength *R*_*kE*_ > 0 and the inhibitory connection strength *R*_*kI*_ < 0. Since each neuron is expected to receive projections from *K* pre-synaptic excitatory neurons and *K* pre-synaptic inhibitory neurons, sparse connectivity is reflected by the assumption that *K* ≪ *N*_*E*_, *N*_*I*_. In advancing the model dynamics for each neuron, the mean time between subsequent updates is τ_*E*_ = 10 ms for excitatory neurons and τ_*I*_ = 9 ms for inhibitory neurons, reflecting experimental estimates of cortical membrane potential time constants (McCormick et al., 1985; van Vreeswijk and Sompolinsky, 1996; Shelley et al., 2002). Based on the total synaptic drive at each time the system is updated, a given neuron is either in a quiescent ($\sigma_{k}^{i}\left(t\right)=0$) or firing ($\sigma_{k}^{i}\left(t\right)=1$) state.

To partition the model across the two subpopulations, the neurons and their corresponding activity variables may also be indexed from *l* = 1, …, *N*, with the first *N*_*E*_ indices corresponding to neurons in the excitatory population and the second *N*_*I*_ indices corresponding to neurons in the inhibitory population. Using this choice of indexing, **R** is the *N*×*N* recurrent connectivity matrix and **p** is the *N*-vector of static external inputs for the network. The external input **p** is selected such that ${\left(\text{F}\text{p}\right)}_{k}^{i}$ is $O\left(\sqrt{K}\right)$ for each neuron, thereby comparable to the total synaptic drive from each population. Analogously, the feed-forward connectivity matrix **F** is *N* × *N* and diagonal, such that diagonal entries *F*_*ii*_ = *f*_*E*_ for *i* = 1, …, *N*_*E*_ and *F*_*ii*_ = *f*_*I*_ for *i* = *N*_*E*_+1, …, *N*_*E*_+*N*_*I*_, thereby scaling the relative external input strength for each respective population. Since the absolute scale of the neuronal input is inconsequential in this non-dimensional model, we assume connectivity parameters *R*_*EE*_ = *R*_*IE*_ = 1, so the primary parameters that determine the inhibition relative to excitation are the post-synaptic connection strengths for the inhibitory neurons and the external input strengths.

Since *R*_*kl*_ as well as θ_*k*_ are O(1) and the external drive is $O\left(\sqrt{K}\right)$, if the excitatory and inhibitory inputs are not balanced, the total synaptic drive is $O\left(\sqrt{K}\right)$ and thus each neuron either fires with an excessively high rate or remains nearly quiescent. In the balanced operating regime, however, the excitatory and inhibitory inputs instead dynamically cancel and produce physiological firing dynamics, leaving the mean synaptic input nearly vanishing with relatively large O(1) input fluctuations responsible for the exact timing of firing events and their irregular distribution. This leads to theoretical conditions on the connection strength parameters (van Vreeswijk and Sompolinsky, 1996, 1998):

(3) $$\begin{array}{r} \frac{f_{E}}{f_{I}}>\frac{R_{EI}}{R_{II}}>1. \end{array}$$

**Figure 1:**
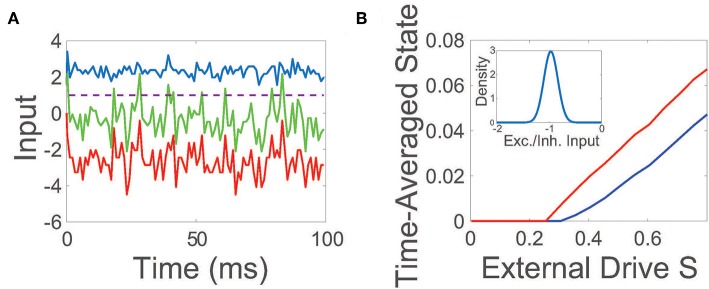
Balanced network dynamics. **(A)** Excitatory (blue), inhibitory (red), and net (green) inputs into a sample excitatory neuron in a balanced network. The dashed line indicates the firing threshold. **(B)** The time-averaged state of the excitatory (blue) and inhibitory (red) population as a function of external input scaling strength *S*. Inset: Probability density of the time-averaged ratio of excitatory and inhibitory inputs across the network. Unless otherwise specified, parameters utilized are *R*_*EE*_ = *R*_*IE*_ = 1, *R*_*II*_ = −1.8, *R*_*EI*_ = −2, *N*_*E*_ = 800, *N*_*I*_ = 200, *f*_*E*_ = 1.2, *f*_*I*_ = 1, *K* = 0.03*N*_*E*_, θ_*E*_ = 1, and θ_*I*_ = 0.7. The external drive into a neuron in the *k*^th^ population is an excitatory constant current that is independently generated and identically uniformly-distributed with $O\left(\sqrt{K}\right)$ magnitude scaled by *f*_*k*_.

The net input into a representative neuron in the balanced state is plotted in Figure 1A, demonstrating a dynamic tracking of excitatory and inhibitory inputs such that the mean total input is far below threshold. On the larger scale of the entire network, an equilibrium between excitation and inhibition is also achieved in the balanced regime, with the time-averaged mean of the ratio between the excitatory and inhibitory input (E/I input ratio) across the network narrowly distributed near −1.

While the sparsity of **R** in principle reduces the necessary data for a successful reconstruction of the network connectivity, compressive sensing theory generally only applies to the recovery of sparse inputs into linear and time-invariant systems (Candes et al., 2006; Donoho, 2006), rather than from measurements of the non-linear and time-evolving dynamics of a neuronal network. To overcome this theoretical challenge, it is important to note that for a broad class of physiological neurons as well as realistic neuron models, the neuronal firing activity exhibits linear dependence on relatively strong external inputs in the proper dynamical regime (Brunel and Latham, 2003; Rauch et al., 2003; Fourcaud-Trocmé and Brunel, 2005; La Camera et al., 2006; Barranca et al., 2014a). Considering the dynamic balance between excitatory and inhibitory inputs facilitates a rapid and robust linear response to external inputs (van Vreeswijk and Sompolinsky, 1996, 1998), we hypothesize that balanced neuronal network dynamics are critical to the efficient CS reconstruction of sparse network connectivity.

For the binary-state balanced network model, the temporal expectation of Equation (2) yields a natural linear input-output mapping in response to a single input vector ***p***

(4) $$\begin{array}{r} \mu =\text{R}\text{x}+\text{F}\text{p}, \end{array}$$

where ***μ*** is an *N*-dimensional vector denoting the time-averaged total input into each neuron and **x** is an *N*-dimensional vector denoting the time-averaged state of each neuron.

To demonstrate the generality of our network reconstruction framework with respect to external inputs and to avoid specializing their design, we drive the network with an ensemble of *r* random input vectors with independent identically uniformly distributed elements, denoted by ${\left\{{\text{p}}^{\left(i\right)}\right\}}_{i=1}^{r}$, and measure the evoked time-averaged net input and state of the neurons, denoted by ${\left\{\mu^{\left(i\right)}\right\}}_{i=1}^{r}$ and ${\left\{{\text{x}}^{\left(i\right)}\right\}}_{i=1}^{r}$, respectively, over a short time duration. From a physiological standpoint, on a given trial, we inject into each neuron a distinct constant current of magnitude determined by a uniformly distributed random variable and measure the evoked dynamics across the network, subsequently reconstructing the network connectivity from a linear mapping relating these quantities. To facilitate efficient recovery, the number of trials utilized *r* ≪ *N*^2^. Here the *N*^2^ entries of **R** are to be recovered using only *Nr* state measurements, leading to a highly underdetermined inverse problem. However, since **R** is sparse, CS may still potentially yield a successful reconstruction (see the Methods section for details).

While conventional balanced network theory assumes a constant and homogeneous excitatory external input is injected into each population (van Vreeswijk and Sompolinsky, 1996, 1998), note that we choose the excitatory external input vector **p** to be composed of independent and identically distributed random variables. Even for these heterogeneous external inputs, balanced dynamics are still well-maintained under population scalings with *f*_*E*_ > *f*_*I*_. The maintenance of balance across the majority of the network can be seen in the inset of Figure 1B, plotting the mean E/I input ratio across the network, which closely resembles the distribution for the $O\left(\sqrt{K}\right)$ constant homogeneous input case in its narrow peak near −1 (van Vreeswijk and Sompolinsky, 1996, 1998; Barranca et al., 2019). To further probe the evoked network dynamics, we empirically examine the response of the network to increasingly large random external inputs in Figure 1B, adjusting scaled external input *S***Fp** by increasing the scaling strength *S*. We observe that as the external drive strength is increased, the time-averaged state of both the excitatory and inhibitory populations intensifies linearly with *S* for sufficiently large inputs, thereby demonstrating linear gain in agreement with Equation (4) and as expected theoretically in the large network limit in the case of homogeneous external inputs (van Vreeswijk and Sompolinsky, 1996, 1998).

With linear input-output mapping (4), we obtain a system of equations relating the network input, evoked dynamics, and the connectivity structure of **R**. To recover the *i*^th^ row of **R** in this case, denoted **R**_i*_, it is necessary to utilize the full set of inputs, $P=\left[p^{\left(1\right)}\ldots p^{\left(r\right)}\right]$, the respective time-averaged inputs into the *i*^th^ neuron, ${\text{U}}_{i}=\left[\mu_{i}^{\left(1\right)}\ldots \mu_{i}^{\left(r\right)}\right]$, and the respective evoked time-averaged states of the *i*^th^ neuron, ${\text{X}}_{i}=\left[x_{i}^{\left(1\right)}\ldots x_{i}^{\left(r\right)}\right]$.

The resultant underdetermined linear system in recovering the *i*^th^ row, **R**_i*_ of the recurrent connectivity matrix is

(5) $$\begin{array}{r} {\text{R}}_{i*}\text{X}={\text{U}}_{i}-{\left(\text{F}\text{P}\right)}_{i*}. \end{array}$$

Since **R** is sparse and the respective average states in **X** are approximately uncorrelated in the balanced regime (van Vreeswijk and Sompolinsky, 1996, 1998), the optimal row reconstruction is the solution to Equation (5) with minimal *L*_1_ norm (Candes et al., 2006; Donoho, 2006) in accordance with CS theory. Considering the resultant *L*_1_ minimization problem is solvable in polynomial time (Donoho and Tsaig, 2008) and since Equation (5) represents a sequence of independent linear systems with respect to the row index *i*, parallelization furnishes a computationally efficient reconstruction of **R**.

**Figure 2:**
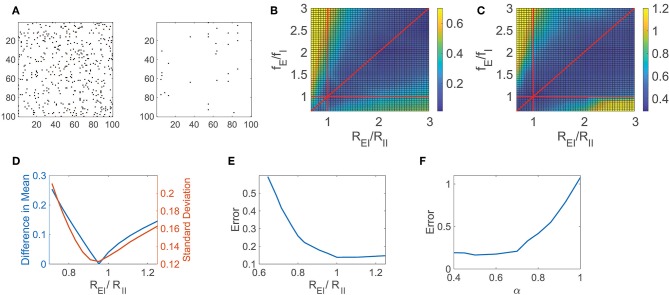
CS network reconstruction and dynamical regime. **(A)** The connectivity matrix **R** for a 100 excitatory neuron subset of a balanced network with *N*_*E*_ = 800, *N*_*I*_ = 200, and 0.05 connection density is depicted on the left. Existing connections are marked in black. On the right, errors in the CS reconstruction of **R** are marked in black. The relative reconstruction error is ϵ = 0.14. **(B)** Difference in absolute value between the mean of the time-averaged ratio of excitatory and inhibitory input across the network (E/I input ratio) and −1, the value expected in the balanced state, as a function of the quotients *R*_*EI*_/*R*_*II*_ and *f*_*E*_/*f*_*I*_. **(C)** Relative reconstruction error of **R** as a function of *R*_*EI*_/*R*_*II*_ and *f*_*E*_/*f*_*I*_. In **(B,C)**, red lines denote *R*_*EI*_/*R*_*II*_ = 1, *f*_*E*_/*f*_*I*_ = 1, and *R*_*EI*_/*R*_*II*_ = *f*_*E*_/*f*_*I*_. **(D)** Statistics of the E/I input ratio across the network as a function of *R*_*EI*_/*R*_*II*_ for fixed *f*_*E*_/*f*_*I*_ = 1.2. Left ordinate axis: Difference in absolute value between the mean *E*/*I* input ratio and −1, Right ordinate axis: Standard deviation of *E*/*I* input ratio. **(E)** Relative reconstruction error of **R** as a function of *R*_*EI*_/*R*_*II*_ for fixed *f*_*E*_/*f*_*I*_ = 1.2. **(F)** Relative reconstruction error of **R** as a function of the exponent α. Each reconstruction utilizes *r* = 900 inputs with 2.5 s observation time.

In Figure 2A, we consider a sparsely connected network with balanced dynamics and 0.05 connection density, and reconstruct its connectivity matrix composed of *N*^2^ = 10^6^ entries using Equation (5) for *i* = 1, …, *N*, recording the network response to *r* = 900 random inputs for 2.5 s each. The connectivity matrix for a subset of 100 excitatory neurons is depicted alongside the corresponding reconstruction error, demonstrating that the majority of connections, or lack thereof, are indeed captured. Improving significantly upon preexisting approaches for reconstructing network connectivity, which commonly require long observation times and focus primarily on excitatory networks (Timme, 2007; Eldawlatly et al., 2010; Hutchison et al., 2013; Zhou et al., 2013b; Goñi et al., 2014), this reconstruction framework successfully distinguishes between excitatory and inhibitory connection types over short observation times. Since the neuron types are not assumed to be known *a priori*, we note that while there is no constraint that excitatory and inhibitory connections are of the appropriate sign directly enforced in solving optimization problem (5) via *L*_1_ minimization, with sufficiently rich measurements of the network dynamics, the connectivity reconstructions nevertheless are generally able to successfully identify both connection signs and magnitudes, as indicated by the small relative error obtained in recovering the connectivity matrix.

To quantify the accuracy of the entire connectivity matrix reconstruction, **R^recon^**, we measure the relative reconstruction error, ϵ = ∥**R**−**R**^recon^∥/∥**R**∥, using the Frobenius norm, $∥\text{R}∥=\sqrt{\underset{i}{\sum }\underset{j}{\sum }R_{ij}^{2}}$. In this particular case, utilizing significantly less trials than entries in **R**, the network relative reconstruction error is only ϵ = 0.14, yielding close agreement with the original connection matrix. We remark that in this network the ratio of excitatory to inhibitory neurons is chosen to be 4:1 in agreement with estimates in the primary visual cortex (Gilbert, 1992; Liu, 2004; Cai et al., 2005; Zhou et al., 2013a), though this framework is adaptable to other distributions of neuron types corresponding to alternative cortical regions. While in this work we specifically consider the role of balanced dynamics in the context of an analytically tractable binary-state model setting, the compressive sensing reconstruction framework naturally generalizes to alternative model networks. In the case of the integrate-and-fire model (Lapicque, 1907; Burkitt, 2006; Mather et al., 2009; Barranca et al., 2014a), for example, rather than requiring detailed knowledge of the networks' inputs as in the binary-state model, the network input-output mapping may instead involve the time-averaged neuronal membrane potentials and firing rates (Barranca et al., 2014b), yielding a framework that is more amenable to experimental settings.

### 2.2. Balanced Network Characteristics for Optimal Reconstruction

We posit that the network functioning in the balanced operating regime is fundamental to the success of the CS reconstruction and demonstrate that the relative reconstruction error indeed increases as the network departs from the balanced state. We confirm the central role of the balanced state in network reconstruction by varying several network connectivity parameters, which crucially determine the network operating state, and examining the resultant impact on the CS reconstruction of **R**.

For the network dynamics to be appropriately balanced in the large *K* limit, Equation (3) gives restrictions on the external and cortical input strengths for the network. These parameter restrictions hold approximately for the sparsely-connected networks of large yet finite size that we examine, and we analyze the impact of these parameters on the network reconstruction accuracy. Since we are considering the connectivity reconstruction for networks composed of a finite number of neurons and therefore Equation (3) only holds approximately, in many cases the dynamics may be well-balanced even though the corresponding theoretical condition in the large network limit is violated (van Vreeswijk and Sompolinsky, 1996; Gu et al., 2018). For this reason, to gauge the degree to which a finite-sized network exhibits balanced dynamics, we analyze the absolute difference between the mean E/I input ratio for all neurons and −1, the expected value for balanced dynamics, as depicted in Figure 2B across network parameters. Here we vary the quotients, *R*_*EI*_/*R*_*II*_ and *f*_*E*_/*f*_*I*_, which are each crucial to Equation (3), observing a clear region of well-balanced dynamics. Investigating the impact of the network dynamical regime on the CS reconstruction of **R**, we plot in Figure 2C the corresponding relative reconstruction error over the same parameter space. The highest quality reconstructions are generally achieved when the mean E/I input ratio is near −1, and the network is consequently in the balanced operating regime, with degradation in accuracy incurred as the mean E/I input ratio departs from −1.

Similarly, we examine a detailed one-dimensional slice of these plots in Figures 2D,E, respectively, as we fix *f*_*E*_/*f*_*I*_ = 1.2 and vary the quotient *R*_*EI*_/*R*_*II*_. In particular, we plot the absolute difference between the mean E/I input ratio and −1 as well as the standard deviation of the E/I input ratio across the network in Figure 2D to further classify the network operating state. We observe that when Equation (3) is approximately satisfied, the difference between the mean *E*/*I* input ratio and −1 is small. In this same regime, the standard deviation of the *E*/*I* input ratio is also near zero, indicating a dynamic balance between the excitatory and inhibitory inputs over the entire network. For nearly identical parameter choices as those producing balanced dynamics, we observe that the corresponding relative reconstruction error, depicted in Figure 2E, is minimal. As the reconstruction accuracy diminishes, increasingly large proportions of neurons remain either active with unrealistically high firing rate or are completely quiescent. Since the relatively rare and irregular threshold crossings due to input fluctuations in the balanced regime largely reflect the impact of the network connectivity on dynamics, nearly frozen or excessively high neuronal activity results in significantly diminished reconstruction quality.

Another crucial assumption in formulating the balanced network model is strong synapses. Similar models could be formulated with connection strengths of form $R_{kl}/K^{\alpha }$. However, the dynamics are only well-balanced in the large *K* limit for α = 1/2. For 1/2 < α ≤ 1, the weaker synapse case, the temporal input fluctuations decrease with *K*, scaling as *K*^1/2−α^, leading to mean-driven dynamics in the large *K* limit. In contrast, for 0 < α <1/2, the stronger synapse case, input fluctuations instead grow with *K*, and thus the net input wildly fluctuates well above and below threshold.

Using our CS framework to reconstruct the network connectivity **R**, we examine the reconstruction error achieved for the network model initialized across choices of α in Figure 2F while fixing *K* and the remaining model parameters. The optimal reconstruction is achieved near α = 0.5, when the network is in the balanced operating regime, with error generally increasing as α moves away from 0.5 and the mean *E*/*I* input ratio deviates from balance. Note that while here we study the impact of α for network realizations with a fixed and finite choice of *K*, the theoretical considerations in the large network limit suggest that these effects become more pronounced for larger networks with correspondingly larger *K*. Considering that the reconstruction error increases especially rapidly as α → 1, we hypothesize that weaker synapses in non-balanced network models are not conducive to the reconstruction of network connectivity, particularly in the mean-field limit. While mean-driven dynamics generally well encode information regarding network inputs and feed-forward connectivity (Barranca et al., 2016b), in this case, we instead observe that the balanced dynamical regime is better suited for encoding recurrent interactions in the network dynamics.

### 2.3. Robustness of Connectivity Reconstruction

**Figure 3:**
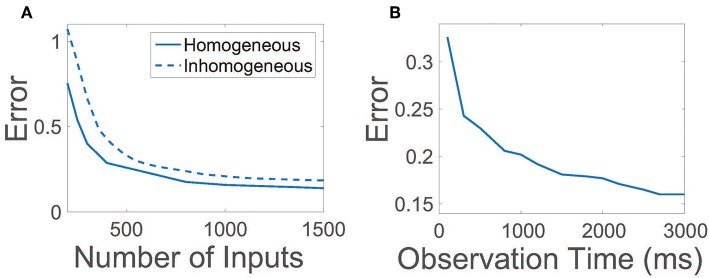
Efficiency and robustness of CS network reconstruction. **(A)** Relative reconstruction error as a function of the number of random input vectors *r* utilized. Solid line depicts error using homogeneous thresholds θ_*E*_ = 1, θ_*I*_ = 0.7. Dashed line depicts error using inhomogeneous thresholds such that $\theta_{k}^{i}=\theta_{k}+\delta_{k}^{i}$, where inhomogeneities $\delta_{k}^{i}\in \left(0,d\right)$ are uniformly distributed random variables and *d* = 0.3. In each case, the observation time is 2.5 s. **(B)** Relative reconstruction error as a function of observation time. In each case, 900 random input vectors are utilized.

For efficiency, it is desirable to achieve an accurate reconstruction of the network connections using a relatively small number of random inputs and also by collecting the evoked network activity over a small observation time. In Figure 3A, we plot the relative reconstruction error for **R** as the number of input vectors is increased given a fixed observation time. Initially, as the number of inputs is increased, the error rapidly decreases. Once the number of inputs utilized is sufficiently large, near ~800, more marginal improvements are garnered, at which point additional experiments are of less utility. Hence, only a relatively small number of trials are necessary to yield near-maximum reconstruction quality.

Given a sufficient number of inputs such that the reconstruction error saturates, we next examine the duration of time over which data must be recorded for successful connectivity reconstruction in Figure 3B. The relative reconstruction error precipitously drops for small observation times, leveling off for sufficiently large time durations over 2 s. Thus, for each set of inputs utilized, it is only necessary to record neuronal activity over a short time duration.

Similar dependence on input ensemble size and observation time holds for networks of alternative sizes with analogous connection density and architecture, yielding comparably accurate reconstructions by using a relatively small number of input vectors. As the irregular dynamics of neurons in the balanced state is crucial to the success of the CS recovery framework, we note that regardless of the observation time and number of inputs utilized, reconstruction of **R** remains intractable if the network dynamics are not sufficiently well-balanced.

In our original binary-state model, we had assumed that all excitatory neurons and inhibitory neurons are statistically homogeneous. We now examine the effect of inhomogeneity in the network on the reconstruction of **R** by varying the firing threshold for each neuron. In this case, thresholds are chosen such that the firing threshold for the *i*th neuron in the *k*th population is $\theta_{k}^{i}=\theta_{k}+\delta_{k}^{i}$, with inhomogeneities prescribed by identically uniformly distributed random variables $\delta_{k}^{i}\in \left(0,d\right)$. In Figure 3A, we plot the reconstruction error dependence on the number of random inputs for inhomogeneity strength *d* = 0.3 ≈ 0.43θ_*I*_, observing only a minor degradation in reconstruction quality relative to the homogeneous threshold case. Thus, we expect that even if a network is composed of neurons of many types, as long as the neuronal dynamics are robustly balanced, it is possible to still utilize our CS framework to reconstruct the network connectivity.

## 3. Discussion

Addressing the current theoretical and experimental difficulties in measuring the structural connectivity in large neuronal networks, we show that the high degree of sparsity in network connections makes it feasible to accurately reconstruct network connectivity from a relatively small number of measurements of evoked neuronal activity via CS theory. The success of this reconstruction depends on the dynamical regime of the network, with the balanced operating state facilitating optimal recovery. Just as the connectivity matrix **R** may be recovered from dynamical activity based on an underlying linear mapping, such as Equation (4), unknown network feed-forward connectivity as well as natural stimuli may analogously be reconstructed (Barranca et al., 2016b). We have empirically verified such reconstructions are also improved when the network is in the balanced operating regime. In light of this, we hypothesize that evolution may have fine-tuned much of the cortical network connectivity to optimize both the encoding of sensory inputs as well as local connectivity based on balanced network dynamics.

It is important to note that while the compressive sensing theory leveraged in this work is well-suited for the reconstruction of sparse signals, the reconstruction of densely-connected neuronal networks in the brain with potentially strongly correlated dynamics remains a challenging area for future investigation (Wang et al., 2011a; Markov et al., 2013; Yang et al., 2017). Though we considered a balanced network model with statistically homogeneous random connectivity among neuron types, physiological neuronal circuits observed in experiment typically exhibit a complex network structure (Massimini et al., 2005; Bonifazi et al., 2009; Markov et al., 2013), which may induce stronger correlations and oscillations in neuronal dynamics (Honey et al., 2007; Wang et al., 2011a; Yang et al., 2017). While prolonged synchronous dynamics may make it infeasible to reconstruct network connections using our methodology, intermixed periods of irregular dynamics may provide sufficient neuronal interaction data or, otherwise, connections between functional modules may be potentially identifiable. Recent theoretical analysis demonstrates that even for networks with small-world or scale-free structure, balanced dynamics can persist in these neuronal networks with various types of single-neuron dynamics, particularly in an embedded active core of neurons hypothesized to play a key role in sparse coding (Gu et al., 2018). For such a balanced core in a network with heterogeneous connectivity, the primary dynamical assumptions of our reconstruction framework hold as does compressive sensing theory in the presence of mildly structured sampling matrices (Elad, 2007; Barranca et al., 2016a; Adcock et al., 2017), and thus it may be possible to extend our framework in recovering connections within the balanced core.

While this work utilized specific modeling choices for which the balanced state is well-characterized, in alternative settings, a similar framework can potentially be utilized to reconstruct sparse network connectivity as long as the dynamics are in the balanced operating regime. Linear mappings in the balanced state similar to Equation (4) have been well-established for various classes of neuronal network models, including those with more physiological dynamics (Brunel and Latham, 2003; Fourcaud-Trocmé and Brunel, 2005; Barranca et al., 2014a, 2019; Gu et al., 2018), and experimental measurements of neuronal firing-activity also generally exhibit a similar linear dependence on input strength (Rauch et al., 2003; La Camera et al., 2006). Advances in multiple neuron recording, such as multiple-electrode technology, optical recording with fast voltage-sensitive dyes, and light-field microscopy, have facilitated the recording of increasingly large numbers of neurons simultaneously (Stevenson and Kording, 2011; Prevedel et al., 2014; Frost et al., 2015), and combined with new optogenetic as well as optochemical techniques for precisely stimulating specific neurons (Banghart et al., 2004; Rickgauer et al., 2014; Packer et al., 2015), we expect the theoretical framework developed to be generalizable by combining these techniques in experiment. To circumvent potential experimental difficulties in simultaneously stimulating specific neurons and recording their evoked dynamics, we expect it to be also possible to extend our theoretical framework by driving a subset of neurons and recording the response of a random group of neurons in each trial. Since a particular subnetwork of neurons in the brain generally receives inhomogeneous and unknown input from external neurons, the development and utilization of an accurate input-output mapping involving only the recorded network dynamics and applied drive in experiments marks a key area for future exploration. While there are known mappings that make no assumption of the detailed input into each neuron, they do assume that external inputs are fully characterized. Since such mappings are quite robust in the presence of noise (Barranca et al., 2014b,c, 2016b), it may still be possible to well discern recurrent connections even in the presence of unknown external neuronal inputs for sufficiently strong forcing applied in experiments.

## 4. Methods

### 4.1. Compressive Sensing Theory

Compressive sensing theory states that for sparse data, the number of measurements required for a successful reconstruction in a static and linear system is determined by the number of dominant non-zero components in the data (Candes et al., 2006; Donoho, 2006). Using this reasoning, optimally reconstructing sparse data from a small number of samples requires selecting the sparsest reconstruction consistent with the measured data, since such a signal is most compressible. CS theory thus provides a significant improvement in sampling efficiency from the conventional Shannon-Nyquist theorem, which asserts that the sampling rate should instead be determined by the full bandwidth of the data (Shannon, 1949).

The reconstruction of time-invariant data from a small number of samples in a linear system can be considered an underdetermined inverse problem. For an *n*-component signal, **y**, *m* discrete samples of **y** can be represented by **Ay**, where **A** is an *m*×*n* measurement matrix composed of rows which are each a set of measurement weights. This yields an *m*-component measured signal, **b**. Reconstructing the true data **y** from the measured data **b** is therefore equivalent to solving

(6) $$\begin{array}{r} \text{Ay}=\text{b}. \end{array}$$

When the number of samples taken is significantly smaller than the number of components in **y**, i.e., *m* ≪ *n*, the above system is highly underdetermined with an infinite number of possible solutions. While one approach to selecting the most compressible solution is to choose the sparsest **y** satisfying Equation (6), this is generally too computationally expensive for real-world signals.

For sufficiently sparse *y* and a broad class of measurement matrices, CS theory shows that a viable surrogate is in fact minimizing $\left|y{|}_{{\text{L}}_{1}}=\overset{n}{\underset{i=1}{\sum }}|y_{i}\right|$ (Candes and Wakin, 2008), which is efficiently solvable in polynomial time using numerous algorithms (Tropp and Gilbert, 2007; Donoho and Tsaig, 2008). From an experimental standpoint, it is relatively straightforward to devise sampling schemes such that the corresponding measurement matrices are amenable to CS. Measurement matrices exhibiting randomness in their structure are particularly viable candidates (Baraniuk, 2007; Candes and Wakin, 2008; Barranca et al., 2016a), and, consequently, the response matrix **X** in the left-hand side of Equation (5) is suited for CS reconstructions in the balanced regime since **X** demonstrates little correlation among its entries.

## Data Availability Statement

All datasets generated for this study are included in the manuscript/supplementary files.

## Author Contributions

All authors listed have made a substantial, direct and intellectual contribution to the work, and approved it for publication.

### Conflict of Interest

The authors declare that the research was conducted in the absence of any commercial or financial relationships that could be construed as a potential conflict of interest.
